# Frequency and Determinants of Breastfeeding in Greece: A Prospective Cohort Study during the COVID-19 Pandemic

**DOI:** 10.3390/children9010043

**Published:** 2022-01-02

**Authors:** Maria Tigka, Dimitra Metallinou, Christina Nanou, Zoi Iliodromiti, Katerina Lykeridou

**Affiliations:** 1 Department of Midwifery, University of West Attica, 12243 Athens, Greece; maria.tigka@gmail.com (M.T.); nanouxv@uniwa.gr (C.N.); klyker@uniwa.gr (K.L.); 2 Department of Obstetric Emergency, General and Maternity Hospital “Helena Venizelou”, 11521 Athens, Greece; 3 Department of Neonatology, Medical School, National and Kapodistrian University of Athens, Aretaieio Hospital, 15772 Athens, Greece; ziliodromiti@yahoo.gr

**Keywords:** breastfeeding frequency, breastfeeding determinants, COVID-19 pandemic

## Abstract

Breastfeeding is considered to be the cornerstone of child health. In Europe however, overall breastfeeding rates remain low. The present study aimed at estimating the frequency of breastfeeding in Greece during the COVID-19 pandemic period and comparing findings with the latest national study in order to identify a potential impact of the pandemic. Additionally, possible correlations of socio-cultural and demographic characteristics with breastfeeding indicators were investigated. This prospective cohort study included 847 women from five tertiary maternity hospitals, between January and December 2020. Data were collected by a structured questionnaire via interview during hospitalization and via telephone in the first, third and sixth month postpartum. Results showed that all breastfeeding indicators improved over the last three years. Full breastfeeding reached 7.2%, contrary to 0.78% of the latest national study at six months postpartum. Employment, marital status, educational level, mode of delivery, type of maternity hospital, body mass index before pregnancy, previous breastfeeding experience of the mother and infant’s birth weight correlated significantly with breastfeeding indicators at different time periods. The COVID-19 pandemic seems to have favorably influenced breastfeeding initiation and duration in Greece due to lockdowns, home confinement and teleworking.

## 1. Introduction

Breastfeeding is considered to be the cornerstone of child survival and child health, as it provides essential nutritional components and numerous biologically active molecules for a child’s growth, development and immune system. The short and long-term health benefits of breastfeeding for children have been well documented [1,2]; thus, exclusive breastfeeding (EBF) for the first 6 months of life is regarded as the golden standard of infant nutrition from current professional organizations, including the World Health Organization (WHO), the American Academy of Pediatrics (AAP), and the Association of Women’s Health, Obstetric, and Neonatal Nursing [3]. Optimal breastfeeding practices have been associated with a lower risk of childhood gastrointestinal, respiratory and urinary tract infections, otitis media, asthma, allergies and sudden infant death syndrome [2,4]. Research evidence also supports the protective effect of breastfeeding on the incidence of obesity, diabetes mellitus and cardiovascular diseases on both mother and child [4,5]. Women who breastfeed also have a reduced risk for breast and ovarian cancer, osteoporosis, metabolic syndrome, rheumatoid arthritis and postpartum depression [5]. Breastfeeding is ultimately an experience that contributes, beyond a shadow of a doubt, to the bonding relationship of the mother-child dyad [6].

The United Nations International Children’s Emergency Fund (UNICEF) and WHO recommend EBF from within an hour after birth until the infant is 6 months old, but, thereafter, nutritious and safe complementary foods should be introduced to the child’s diet while continuing to breastfeed for up to two years or beyond [7,8]. Globally, low-income and middle-income countries were already close to the World Health Assembly’s initial target of 50% by 2025, as EBF rates for infants younger than 6 months improved from approximately 35% in 2000 to approximately 49% in 2019 [9], a rate which is consistent with the 44% of the UNICEF’s analysis relating global trends in EBF for the years 2014–2020 [10]. On the contrary, upper-middle-income countries reached a rate of 37% in 2019, indicating a struggle to reach the international goal [9]. Thus, much remains to be done to make EBF the norm for infant feeding.

In Europe especially, the overall breastfeeding rates remain low. Between 2006 and 2012, only 25% of the infants in the WHO European Region were exclusively breastfed for the first 6 months, in comparison to the 43% of the infants in the WHO South-East Asia Region [11]. Recent data from 24 European countries showed that 23% of the infants aged less than 6 months were exclusively breastfed, while only 13% of them were exclusively breastfed when they were at 6 months of age. The highest EBF rate was reported in Slovakia (49%) and Hungary (44%) and the lowest in Greece (1%), Finland (1%) and the United Kingdom (1%) [4]. To our knowledge, the latest survey conducted among 11 European countries reported that EBF rates gradually decline over time, leading to a rather low rate of 25% at 6 months of age [12]. Despite the fact that EBF rates varied substantially across these countries, all of them remain way below the global recommendation. However, results that report rates of EBF need to be interpreted with caution, as differences on aspects of definition, research design and methodology are found among the published surveys [13].

Recent data from the Greek research center “Institute of Child Health” (ICH) showed that some slight improvements have been recorded in breastfeeding rates during the last years in Greece. The ICH investigated the potential alterations of breastfeeding trends from 2007 to 2017. The results indicated that any breastfeeding at 6 months was increased from 22% in 2007 to 45% in 2017 and rates of EBF initiation in the first day of life improved around 25%. However, the percentage of EBF at 6 months of age remained very low (0.9% in 2007 and 0.78% in 2017) [14], highlighting the need for further assessment of the causes that locate Greece at the lowest ranking amongst breastfeeding rates. 

Various factors define and have an impact on breastfeeding initiation and duration, including individual and socio-cultural features of the mother, the child and the family, characteristics of the health-care services, institutional policies, public health and social frameworks, as well as advertising and promotion of different feeding practices [15]. The COVID-19 pandemic, however, is a newly introduced factor that affected all patient populations, including breastfeeding mothers. In the beginning of the pandemic period, due to the concern for potential transmission of the infection from the mother to the newborn, there had been recommendations by the AAP and the Centers for Disease Control and Prevention (CDC) for temporary separation of the mother-baby dyad. Nevertheless, revised guidelines eventually promoted direct breastfeeding and the use of expressed breast milk [16], whereas WHO encouraged direct breastfeeding with hygiene preventive measures and recommended the separation of mother and neonate only in cases of poor maternal health conditions or the need to implement therapeutic interventions to the newborn [17]. The conflicting, confusing and ongoing updated recommendations for breastfeeding, together with the fear of possible perinatal transmission of the SARS-CoV-2 virus from mother to infant, might have led to changes of breastfeeding tendencies and probably affected maternal decision-making about breastfeeding. However, puerperal women and their families must be very well informed by healthcare professionals to make a conscious choice based on the information and scientific data available in the literature so far [18].

The aim of the present study was to (a) assess the frequency and determinants of breastfeeding in Greece during the COVID-19 pandemic period of the year 2020, (b) investigate possible correlations of breastfeeding rates with socio-cultural and demographic characteristics and factors and finally, (c) compare findings with the latest national study of breastfeeding in Greece conducted in 2017 in order to identify a potential impact of the COVID-19 pandemic. We hypothesized that the COVID-19 pandemic will have favorably influenced breastfeeding initiation and duration in Greece due to lockdowns, home confinement and teleworking.

## 2. Materials and Methods

### 2.1. Study Design

This is a prospective cohort study conducted during the COVID-19 pandemic. The recruitment period was from January to December 2020. The study was conducted according to the guidelines of the Declaration of Helsinki and approved by the Ethics Committees of the following five tertiary hospitals, three public and two private ones, in Attica, Greece:

- General and Maternity Hospital “HELENA VENIZELOU” (24285/29 October 2019)
- “ATTIKON” General University Hospital (570/1 October 2019)
- “ALEXANDRA” General Hospital (511/20 July 2020)
- “IASO” General Maternity and Gynecology Clinic (30 May 2019)
- “LETO” General, Maternity and Gynecology Clinic (174a/5 June 2019)

All participants provided written informed consent after a complete description of the study.

### 2.2. Sample

Eligible participants were all mothers who had given birth and were inpatients in the postnatal wards during the recruitment period. Mothers should meet the following inclusion criteria: (1) speak and understand the Greek language fluently, (2) good health condition for both mother and infant after birth and (3) have a permanent telephone number so that follow-up communication is feasible. Mothers with neonates who had congenital malformations that interfered with breastfeeding were excluded from the study.

Stratified random sampling was used to improve the precision of the study. The population was divided into non-overlapping groups according to the three 8-h-hospital shifts of a workday (07.00–15.00; 15.00–23.00; 23.00–07.00) and each day of the week. The research team then collected a random sample of population members from within each shift and day of week. For instance, a woman that gave birth on Monday during the morning shift was randomly selected from each hospital. Another woman that gave birth on Tuesday during the morning shift was randomly selected from each hospital, and so on, for every shift and day of week in each hospital. Finally, women that had given birth within all shifts, days of week and hospitals were incorporated in the study. This technique ensured that observations from all relevant strata would be included in the sample.

**Figure 1:**
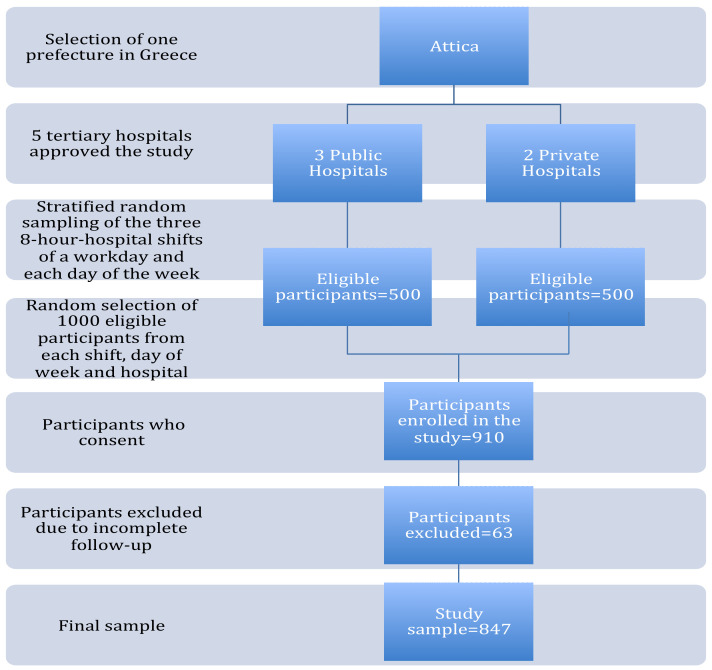
Flowchart of the process of the multistage stratified random sampling method used in the study.

The head midwife of the research protocol informed the eligible mothers of the purpose and the nature of the study. In total, 1000 women were invited to participate. A total of 910 (response rate 91%) agreed to join, but only 847 of them answered completely at all stages of the research process and were taken into account for analysis. The 63 women that could not be reached via telephone communication during the follow-up were excluded from the study due to insufficient answers. The flowchart of the process of the multistage stratified random sampling method used in the study is depicted in Figure 1.

The final sample size was initially determined by specific time and condition constraints, as it was mandatory to complete the recruitment within two months in each maternity hospital. The impact of the recent COVID-19 pandemic on sampling recruitment should not be ignored.

### 2.3. Data Collection

Once the eligible mothers voluntarily agreed to participate, they were firstly given an informed consent form to sign and thereupon the interview for data collection began. The information was gathered from the mothers through a one-to-one structured interview on the 3rd postnatal day. This type of interview was selected in order to (a) provide explanations to the participants when necessary, (b) ensure completeness and clarity of the answers and (c) be quick and easy and therefore achieve future collaboration in the scheduled follow-up.

One, three and six months postpartum, mothers were followed up by phone interviews in order to retrieve relevant information regarding the infant’s feeding patterns, breastfeeding practices and status, as well as the mother’s reasons for breastfeeding cessation. Coding of all participants was automatically created by the database in use, in order to preserve de-identification.

### 2.4. Measurements

Data collection was performed by using a structured questionnaire divided in three sections, designed by the first author for the needs of the research. The selection of the variables included in each section was based on prior knowledge derived from respective studies which had investigated a similar research theme [19,20,21], as well as on our intention to explore further the respective parameters in the Greek setting. The questionnaire was distributed initially to five experts to evaluate its content and check the clarity of the questions. Afterwards, a pilot study in 50 women (who were not enrolled in the present study) was conducted in order to check the degree of understanding of the questions and other features that might need modification. After making the necessary changes, the final form of the questionnaire included both open-ended and closed-ended questions and the baseline interviews typically lasted for about 15 min. Phone call follow-up had an average duration of 5 min.

The first section of the questionnaire included information about maternal demographic and socioeconomic characteristics (age, ethnicity, marital status, employment status, educational level, type of hospital, residential area).

The second section of the questionnaire referred to the obstetrical and lactation history (parity, type of birth, duration of gestation, neonatal sex and birth weight, breastfeeding duration of a previous infant, 6-month breastfeeding follow-up, reason for breastfeeding cessation, maternal pre- and post- pregnancy body mass index (BMI)).

The third section of the questionnaire asked about lifestyle trends (smoking and caffeine consumption before conception, antepartum and postpartum).

### 2.5. Definitions of Breastfeeding

The definitions of breastfeeding used in the present study are according to WHO [7].

- Breastfeeding (BF), hereafter referred to as ‘any breastfeeding’, implies that the child receives breast milk (direct from the breast or expressed). The infant is allowed to receive any food or liquid including non-human milk.
- Exclusive Breastfeeding (EBF) implies that the infant receives only breast milk, including expressed breast milk or milk from a wet nurse, and allows the infant to receive drops and syrups (vitamins, minerals and medicines), but does not allow anything else.
- Predominant Breastfeeding (PBF) implies that the infant receives breast milk (including milk expressed or from wet nurse) as the predominant source of nourishment, and also allows liquids (water, and water-based drinks, fruit juice and oral rehydration solution), ritual fluids and drops or syrups (vitamins, minerals and medicines). Non-human milk and food-based fluids are forbidden when defining PBF.
- Full Breastfeeding (FBF) is defined as exclusive and predominant breastfeeding together.
- Mixed Breastfeeding (MBF) implies that the infant receives breast milk, allowing liquid and non-human milk intake.

In the present study, we also make use of the definition “Breastfeeding without human-milk substitute (BFWHMS)”, in order to determine the percentage of women who breastfeed fully at the age of six months, but whose children have already been introduced to semi-solid/solid foods.

### 2.6. Data Analysis

Qualitative variables are presented as absolute and relative frequencies (%), while quantitative variables are presented using appropriate descriptive statistics (i.e., mean, median, SD, min and max). Chi-square test was implemented to investigate the relationship between qualitative variables. Due to the skewed distribution of our quantitative variables, the Mann–Whitney test or Kruskal–Wallis test was implemented to compare median values between two or more subpopulations, respectively. To investigate factors that affect different types of breastfeeding in various months, multiple logistic regression analysis was used; towards this end, we investigated the effect of various factors (e.g., education, marital status, type of hospital, mode of delivery, BMI before pregnancy, low birth weight of the newborn, duration of previous breastfeeding experience in days, employment at sixth month after delivery, gestational age of the newborn) on the different types of breastfeeding.

A two-sided test with a *p*-value less than 0.05 was considered statistically significant. IBM SPSS v.26 (IBM Corp. Released 2019. IBM SPSS Statistics for Windows, Version 26.0. Armonk, NY, USA: IBM Corp) was used for the statistical analysis.

## 3. Results

### 3.1. Basic Sample Characteristics

Of the 847 women enrolled in this survey, 91.4% of them had Greek nationality. The mean age of all women was 33.7 years (33.7 ± 4.7), while for the primigravida was 32.5 years (32.5 ± 4.7). A high percentage of the studied population was university graduates (47.2%) and employed before pregnancy (80.9%). Approximately half of the participants had given birth in a private hospital. The vast majority of women were married, and almost all the unmarried, were living with a long-term partner, therefore the 99.8% of the whole sample was supported by a partner at home. Almost half of the women were primigravida, the 38.5% were secondgravida and the rest had three or more children (9.2%). Most of the women had a singleton pregnancy and the mean duration of pregnancy was 38.3 weeks (38.3 ± 1.5). The mean maternal BMI was 24.2 kg/m^2^ (24.2 ± 5.0) before pregnancy and 28.6 kg/m^2^ (28.6 ± 4.8) before delivery.

With regard to baseline characteristics related to the infant, our findings revealed a remarkably high percentage of caesarean sections (66.8%). The majority of the infants were full-term, had normal birth weights and almost half of them were males. Maternal baseline sociodemographic characteristics and history related to pregnancy and birth are extensively presented in Table 1 (qualitative variables) and Table 2 (quantitative variables).

### 3.2. Breastfeeding Indicators

**Figure 2:**
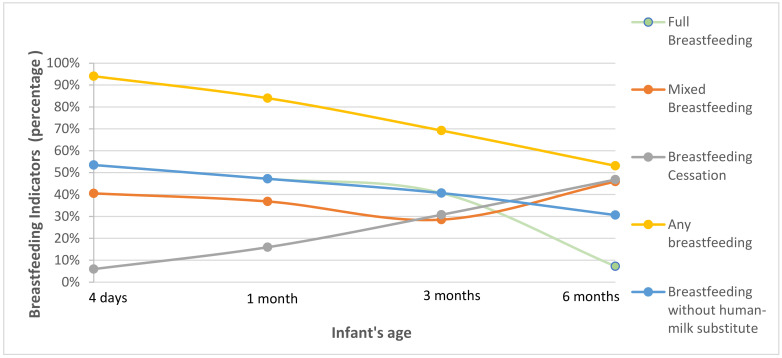
Breastfeeding indicators per time period.

Based on the results of the present study, the following breastfeeding indicators were calculated (Figure 2):

- Any Breastfeeding (ABF): The percentage of infants who were breastfeeding the fourth day of life (usual day of discharge in Greece) was 94.1% and reached 84% at the end of the first month. In the following months, a small drop was observed in the breastfeeding rates, as 69.2% of the infants were still breastfeeding in the third completed month of life. At the end of the six month follow-up, 53.1% of the infants were still breastfeeding.
- Full Breastfeeding: The percentage of infants who were fully breastfed the day of discharge was 53.5% and reached 47.2% at the end of the first month. A gradual decline was observed in the following period, as 40.7% of the infants continued FBF at the third completed month of life. Then, there was a rapid reduction in the rate and the percentage fell to 7.2% at the end of the six month of life.
- Breastfeeding without human-milk substitute: The fourth day of life, 53.5% of the infants were breastfeeding without human-milk substitute. This percentage fell to 47.2% and 40.7% at the end of the first and third month, respectively. At the end of the six month of life, 30.7% of the infants were breastfeeding without receiving or having ever received any human-milk substitute.
- Mixed Breastfeeding: On the fourth day of life, many mothers (40.6%) were breastfeeding and also giving a human-milk substitute to their infants. A decrease in this percentage was observed at the end of the first (36.8%) and third (28.5%) month of life, since some of the women stopped giving a human-milk substitute to their infant and continued with FBF. At the end of the sixth months, however, the percentage of MBF rose again (45.9%), implying that some women started giving a human-milk substitute anew.
- Introduction of solid/semi-solid foods: Information regarding the introduction of solid/semi-solids foods was obtained at the six-month follow-up from 450 women out of the 847 who took part in the study. The number of women who responded was smaller due to the fact that only mothers who continued breastfeeding were contacted at this final follow-up stage. The introduction of solid/semi-solid foods was detected to have started already from the beginning of the fourth month of life (1.7%) and there was a vertical increase in the percentages in the following months. In the fifth month, most of the infants (83.3%) had received solid/semi-solid foods or fruit juice, while the percentage reached 100% in the sixth completed month of life.
- Breastfeeding cessation: As far as breastfeeding cessation is concerned, our results showed that a minority of women (5.9%) had stopped breastfeeding until the fourth postpartum day. This percentage rose to 16%, 30.8% and 46.9% at the end of the first-, third- and sixth- month follow-up, respectively.

The breastfeeding duration of all breastfeeding indicators was 123.88 ± 70.08 (mean ± SD).

### 3.3. Causes of Breastfeeding Cessation

A significant percentage (46.9%) of the studied women ceased breastfeeding within the first six months after birth for various reasons. The most frequent reason for early breastfeeding cessation was the perceived insufficient milk supply (45.3%, Ν = 180/397). From these 180 women, 144 (80%) reported MBF at the day of discharge and 106 (58.9%) had ceased breastfeeding at the third month of the follow-up.

In addition, the 14.4% of women discontinued breastfeeding due to medication intake, either following their physician’s advice or their own choice to do so. An even lower percentage of women (8.6%) decided to stop breastfeeding on their own accord. Difficulty in time management, fatigue and psychological issues led 5.6% of the women to give up breastfeeding, while 4.3% stopped because of the infant’s poor attachment to the breast. Return to work was the reason for 3% of women to cease breastfeeding, whereas 2.8% stopped due to smoking. Women who were not smoking before pregnancy had significantly longer duration of breastfeeding compared to smokers before pregnancy (*p* < 0.001). The remaining causes of breastfeeding cessation reached a percentage equal to 2.5% or less, and are shown extensively in Table 3.

### 3.4. Factors Related to Breastfeeding Indicators

Multiple logistic regression was applied in order to determine the factors that were associated crucially with breastfeeding. Employment and marital status, educational level, mode of delivery, type of maternity hospital, BMI prior to pregnancy and previous breastfeeding experience of the mother, as well as the infant’s birth weight, were factors that correlated significantly with breastfeeding indicators in different time periods (Tables S1–S5).

More specifically, mothers who were not working at six months, when compared to employed ones had an:

1. OR = 5.9 (95% C.I.: 4.1, 8.5), OR = 12.9 (95% C.I.: 8.2, 20.2) and OR = 11.7 (95% C.I.: 2.8, 49.1) to fully breastfeed their infant in the first, third and sixth month postpartum, respectively (*p* ≤ 0.001).
2. OR = 66.6 (95% C.I.: 38.2, 116.1) to follow any breastfeeding at six months postpartum (*p* < 0.001).
3. OR = 31.6 (95% C.I.: 15.8, 63.2) to breastfeed their infant without giving any human-milk substitute at six months postpartum (*p* < 0.001).

In terms of non-employment at six months due to the COVID-19 pandemic, a larger number of women compared to the expected ones, who were on work suspension or teleworking, managed ABF, FBF or BFWHMS at six months postpartum (*p* < 0.001) (Tables S6–S8).

Married women had an OR = 3.5 (95% C.I.: 1.4, 8.6) to fully breastfeed their infants in the third month postpartum as opposed to unmarried ones (*p* = 0.008). Similar results were found in terms of BFWHMS in the sixth month postpartum. This finding highlights that marital status has a crucial role on breastfeeding indicators.

As far as educational level is concerned, the better the mother’s level, the more likely she was to follow any breastfeeding in the sixth month follow-up (Table S4). Additionally, with regard to FBF in the first month postpartum, the same trend was observed with the sole difference that the statistical correlation was stronger for mothers who had university rather than postgraduate studies (Table S1).

Women who had given birth via caesarean section had an OR = 0.6 (95% C.I.: 0.4, 0.8) to fully breastfeed their infant in the first and the third month in comparison to women who had a vaginal delivery. Similar results were found in terms of BFWHMS in the sixth month postpartum. Such results indicate that the mode of delivery is another factor that correlates importantly with breastfeeding indicators.

As for the type of maternity hospital, women who gave birth in a public hospital had an OR = 0.7 (95% C.I.: 0.1, 1.0) to achieve FBF in the first month postpartum, and had an OR = 0.5 (95% C.I.: 0.4, 0.8) to BFWHMS in the sixth month postpartum when compared to women who gave birth in a private hospital.

The BMI of the woman before pregnancy was a factor that was inversely associated with breastfeeding indicators at different time points. The higher a woman’s BMI before pregnancy, the less likely she was to fully breastfeed her infant in the first (OR = 0.9, *p* < 0.001) and third (OR = 0.9, *p* < 0.001) month postpartum; to follow any breastfeeding pattern (OR = 0.9, *p* = 0.01); to breastfeed without giving any human-milk substitute to her infant (OR = 1.0, *p* = 0.007) in the sixth month postpartum.

An additional remarkable result to emerge from our data is that previous breastfeeding experience correlated significantly with breastfeeding indicators. In detail, for each additional day of a mother’s previous breastfeeding experience, we observed an OR = 1.002 (95% C.I.: 1.002, 1.003) to fully breastfeed her infant in the first, third and sixth month postpartum (*p* < 0.001), and OR = 1.002 (95% C.I.: 1.003, 1.006) to follow any breastfeeding at six months postpartum (*p* < 0.001).

Finally, neonate’s low birth weight (LBW) was another factor that was inversely associated with breastfeeding indicators. An LBW infant (less than 2500 gr) had an OR = 0.3 (95% C.I.: 0.1, 0.7) and an OR = 0.3 (95% C.I.: 0.1, 0.8) to be fully breastfed the first (*p* = 0.005) and third (*p* = 0.023) month postpartum, respectively, and an OR = 0.2 (95% C.I.: 0.1, 0.6) to be breastfed without receiving any human-milk substitute at six months postpartum when compared to an infant with birth weight more than 2500 gr.

### 3.5. Comparison of the Present Study with the Latest National Study in Greece

The National Breastfeeding Study conducted by the ICH during the year 2017 [14], is the most recent study that has recorded the alterations on frequency and main determinants of breastfeeding through time in a Greek setting. The ICH is a research center under the oversight and with the funding of the Greek Ministry of Health, whose scope of study and action is research, clinical and laboratory service provision to children and training of professionals working with children. Comparison of the present study with the latest National Breastfeeding Study reveals an improvement of all breastfeeding indicators over the last three years.

More specifically, upon the initiation of breastfeeding and first month follow-up, FBF rates were slightly increased. The FBF rate also remained elevated in the third month postpartum and was much higher than 2017 at 6 months postpartum. The rates of BFWHMS were similar to the FBF ones for the first three months postpartum for both studies. In particular, only in the sixth month postpartum, a higher percentage of women in the present study were breastfeeding without giving any human-milk substitute to their infant, as compared to the latest National Study (30.7% vs. 23.53%). Any breastfeeding rates showed a gradual increase over time when the two studies were compared (Table 4, Figure S1).

## 4. Discussion

The present study, as far as we know, is the first to identify the potential impact of the COVID-19 pandemic on the frequency and determinants of breastfeeding in Greece. The study delivered results that confirmed our initial hypothesis, that is, the COVID-19 pandemic will have positively influenced breastfeeding initiation and duration in Greece due to restrictions, such as lockdowns, home confinement and teleworking.

In comparison with the findings of the latest National Study in 2017 [14], FBF rates were found to have risen by approximately two to eight percentage points for the first three months and reached 7.2% in the sixth month follow-up, contrary to the 0.78% of the previous study. Additional breastfeeding indicators, such as BFWHMS and ABF, were found to have been increased throughout the follow-up period in contradiction with earlier Greek studies [15,22,23]; thus, in our opinion, there are two major possible explanations for these observations. Firstly, two out of the five participating hospitals are certified as ‘breastfeeding-friendly hospitals’ and enhancements in hospital practices, like early skin-to-skin contact, breastfeeding initiation within the first hour after birth and rooming-in, have been found to contribute in favor of breastfeeding rates [14,24]. Secondly, there is an ongoing Greek program named ‘Alkyoni’, which is a national initiative implemented by the ICH with the aim to protect, promote and support breastfeeding through awareness campaigns, educational activities for healthcare professionals and parents and a breastfeeding phone helpline. The aforementioned actions of this program could well be responsible for the increase of breastfeeding rates occurring during the last years in Greece.

Furthermore, it cannot be ruled out that the COVID-19 pandemic had a beneficial effect on breastfeeding rates in the present study. The present study was conducted within 2020, a year included in the COVID-19 pandemic. In December 2019, the first identified cases of COVID-19 were officially reported in Wuhan, China [25] and on the 11th of March 2020, the WHO declared this global health emergency as a pandemic [26]. In Greece, the first case of COVID-19 was identified on the 26th of February 2020 and the Greek government imposed strict containment and social isolation measures (lockdowns) from the 16th of March 2020 onwards. The COVID-19 transmission and protective measures have critically modified typical everyday life, consequently influencing breastfeeding practices as well. Interestingly enough, results from our study, demonstrated that non-employment at six months due to work suspension (10.1%) or teleworking (15.7%) correlated significantly with all breastfeeding indicators, although Latorre et al. [27] recently concluded that lockdowns and home confinement led to a decrease of EBF in non-infected mothers. However, our finding lends support to previous ones in the literature [28,29], where mothers felt that breastfeeding was protected due to lockdowns and delayed return at work. Being more often at home certainly facilitates breastfeeding for some women, while for others it creates more anxiety and stress due to concurrent childcare responsibilities or reduced medical counseling and support [28,29]. Hence, healthcare professionals should not neglect that the personal context and the overall environment at home can affect women’s breastfeeding practices variously. In the future, alternative supportive measures, such as tele-visits by midwives or perinatal organizations, should be considered for all mothers.

It is worth mentioning that, in the present study, the most frequent reason for early breastfeeding cessation was the perceived insufficient milk supply (45.3%), which is in accordance with earlier studies [15,29,30]. Nonetheless, studies investigating milk intake and weight gain in infants being exclusively breastfed have shown that less than 5% of mothers are in fact incapable of producing sufficient milk to meet their infant’s nutritional demands and needs in the first four months of life [15,31]. Such data emphasize the key role of healthcare professionals on promoting, supporting and maintaining breastfeeding [32,33].

Recent Greek studies [14,34] have found caesarean section rates at 53.8% and 58%, whereas, in our study, disappointingly, a much higher percentage of women had delivered via caesarean section (66.8%). A relative observation was that women who had undergone caesarean section correlated negatively with breastfeeding indicators, as they were half as likely to fully breastfeed their infant in the first and third month postpartum, and breastfeed without giving any human-milk substitute in the sixth month postpartum, in comparison with women who had a vaginal delivery. Potentially, the caesarean section can affect adversely the onset of lactation, interrupt mother-infant interaction or constrain infant suckling [35,36]. The unfavorable association between caesarean section and early breastfeeding initiation has been extensively confirmed by other authors [35,36,37].

Sociodemographic characteristics, such as educational level, marital status and employment were correlated with breastfeeding indicators as well, in the present study. The higher educational level was positively associated with breastfeeding in different time periods; a correlation which is consistent with previous results [14,15,37,38,39,40]. More highly educated mothers may enjoy professional benefits, such as flexible work schedule, breastfeeding breaks or a cleaner and safer environment for breastfeeding and pumping, factors which may provide the support needed to breastfeed for a longer time [39]. Moreover, highly educated mothers might choose to breastfeed their infant more consciously. Following on, married women in our study were more likely to fully breastfeed their infants in the third month postpartum as opposed to the unmarried ones. This finding substantiates previous ones in the literature which describe the positive association between marital status and breastfeeding [40,41]. Specifically, the lack of support from the father is correlated to breastfeeding cessation [42], while in other studies such support does not differentiate the prevalence of breastfeeding initiation and duration [23,37]. Finally, mothers in our study who were not working at six months had a greater likelihood to breastfeed their infant in different time periods compared to mothers who were employed at six months. Time off-work after labor enhances the breastfeeding potential [21]. Different factors, such as strict work program, lack of supportive breastfeeding facilities and long distance from workplace, may increase the odds of breastfeeding cessation for employed mothers during the first six months of the infant’s life [21,42,43].

As for the type of maternity hospital, women who gave birth in a public hospital were about half as likely to achieve FBF in the first month postpartum or BFWHMS in the sixth month postpartum when compared to women who gave birth in a private hospital. Iliodromiti et al. [14], however, did not find this factor to have a significant association with breastfeeding. A possible explanation for this finding is that women who give birth in the private sector in Greece usually collaborate with a private midwife continuously during the antenatal period and, therefore, have greater knowledge, more self-efficacy and more positive attitude towards breastfeeding. Midwife-led antenatal breastfeeding counseling and support during the breastfeeding period can lower the perceived breastfeeding barriers during the postpartum period and contribute to an increase of breastfeeding rates [15,32,44].

Other factors that were found to be associated with breastfeeding indicators were the BMI of the mother, the previous breastfeeding experience of the mother and the neonate’s low birth weight. The BMI of the woman before pregnancy was a factor that was inversely associated with breastfeeding indicators at different time points, a finding confirmed by other studies as well [15,38,42]. Additionally, the maternal previous breastfeeding experience was a factor that was found to contribute positively to the initiation and duration of breastfeeding, a finding in agreement with other studies [23,45]. However, prior limited duration of breastfeeding and unfulfilling experience had a negative effect on subsequent breastfeeding [39,45]. Lastly, the neonate’s low birth weight was another factor that was inversely associated with breastfeeding indicators, a finding that was also proposed by other authors [14,15], whereas Brown et al. found no correlation [31].

As far as smoking is concerned, in the present study, we found that women who did not smoke before pregnancy had significantly longer duration of breastfeeding compared to smokers before pregnancy. Maternal smoking is recognized as a negative predictor for breastfeeding [14] and is associated with higher risk for breastfeeding cessation [15]. Therefore, there is an urgent need for smokers to be promptly and vigorously targeted and motivated by healthcare professionals to change lifestyle factors that influence or negate the breastfeeding experience [22]. Remarkably, we did not find a correlation among nationality, maternal age, parity and newborn’s sex with breastfeeding indicators, results that differ from those of Baumgartner et al. [38], Santana et al. [40], Habtewold et al. [41] and Iliodromiti et al. [14], respectively.

This study has gone some way towards enhancing our understanding of the frequency and determinants of breastfeeding during the COVID-19 pandemic, especially in the Greek setting. We are aware, though, that our research had some strengths and limitations. A major strength was that participants came from five tertiary level institutions, not just one, which belong to either the public or private sector and operate as referral centers for mothers of the entire Greek territory, indicating a more representative sample. Although the sample included mothers from the capital and the provinces (22%), it was drawn from a single big city; thus, the generalization of the findings in the whole Greek context is restricted. Additional strengths were the prospective longitudinal design, the stratified random sampling and the high response rates, during both hospitalization and follow-up. As far as breastfeeding indicators are concerned, we investigated FBF rates and not EBF and PBF rates separately, a certain disadvantage of our study that created difficulty in comparing our findings to other studies that reported EBF rates. Finally, the narrow time period that each hospital provided us for sampling recruitment due to COVID-19 restrictions had an undoubted impact on the sample size, which possibly influenced the results obtained, mostly referring to the lack of significant correlations concerning breastfeeding indicators and nationality, maternal age, parity and newborn’s sex.

Despite these limitations, our study suggests some possible directions for future research. Future studies with larger samples from various countries and cultures could assess whether there are differences in frequency and breastfeeding indicators due to the COVID-19 pandemic and investigate additional correlations focused more on clinical factors associated with SARS-CoV-2 and breastfeeding. It would also be of great interest to explore a probable correlation among maternal mental health disorders because of lockdowns and home confinement, and breastfeeding practices and rates.

## 5. Conclusions

The results of the present study suggest an increasing trend in all breastfeeding indicators in Greece during the COVID-19 pandemic, proposing that COVID-19 restrictions have favorably influenced breastfeeding initiation and duration. However, Greece is still far beyond the adoption of the WHO target of EBF rate during the first six months of life and, besides, introduction of human milk substitutes in the early infancy and early introduction of solids after the fourth month of age is thought to be the ‘norm’. These observations demonstrate that healthcare professionals have not promoted and supported breastfeeding in the Greek setting in an effective manner. Implementation of various policy frameworks, initiatives, and continuing educational programs targeted to healthcare professionals and mothers could contribute to the alteration of the current infant feeding practices in Greece.

## Figures and Tables

**Table 1:** Maternal baseline sociodemographic characteristics and history related to pregnancy and birth (qualitative variables).

Variable	Categories	*N* (%)
Nationality	Greek	774 (91.4)
	Other	73 (8.6)
Education	None/Elementary	8 (0.9)
	Junior High/High school	155 (18.3)
	College	142 (16.8)
	University	400 (47.2)
	Postgraduate studies	142 (16.8)
Marital status	Married	808 (95.4)
	Single	39 (4.6)
Living with a partner	Yes	845 (99.8)
	No	2 (0.2)
Residence	Attica	661 (78.0)
	Provinces	186 (22.0)
Employment before pregnancy	Yes	685 (80.9)
	No	162 (19.1)
Employment at sixth month after delivery	Yes	306 (36.1)
	No	541(63.9)
	Work suspension due to COVID-19 pandemic	85 (10.1)
	Teleworking due to COVID-19 pandemic	133 (15.7)
	Maternity leave	152 (17.9)
	Not employed	171 (20.2)
Type of hospital	Private	464 (54.8)
	Public	383 (45.2)
Gravida	Primigravida	443 (52.3)
	Secondgravida	326 (38.5)
	Multigravida	78 (9.2)
Mode of delivery	Vaginal birth	281 (33.2)
	Caesarean section	566 (66.8)
Type of pregnancy	Singleton	834 (98.5)
	Twin	13 (1.5)
Newborn’s sex	Female Male	414 (48.2) 445 (51.8)
Gestational age of the newborn	Preterm < 34 weeks Late preterm 34–36^+6^ weeks Term 37–41^+6^ weeks	17 (2.0) 66 (7.7) 776 (90.3)
Birth weight of the newborn < 2500 gr	Yes No	64 (7.5) 795 (92.5)

**Table 2:** Maternal baseline sociodemographic characteristics and history related to pregnancy and birth (quantitative variables).

Variable	Median (Min, Max)	Mean ± SD	*N*
Age (all women)	34.0 (17.0, 44.0)	33.7 ± 4.7	847
Age (primigravida)	33.0 (17.0, 44.0)	32.5 ± 4.7	443
Duration of pregnancy (weeks)	38.4 (30.0, 42.0)	38.3 ± 1.5	847
BMI before pregnancy	22.9 (15.9, 50.7)	24.2 ± 5.0	847
BMI before delivery	27.7 (19.0, 54.7)	28.6 ± 4.8	847
Birth weight of the newborn	3160.0 (1290.0, 4900.0)	3134.4 ± 451.2	859
Breastfeeding duration (days)	180 (0, 181)	123.88 ± 70.08	847
No Smoking before pregnancy—Breastfeeding duration	180 (0, 181)	130.17 ± 67.13	581
Smoking before pregnancy (Yes)—Breastfeeding duration	135 (0, 181)	110.12 ± 74.43	266
Smoking before pregnancy (Total)—Breastfeeding duration	180 (0, 181)	123.88 ± 70.08	847

**Table 3:** Causes of breastfeeding cessation.

	*n*	%
Breast-related causes		
Perceived low milk quantity	180	45.3
Nipple soreness and cracking- Mastodynia	9	2.3
Mastitis	10	2.5
Flat nipples	6	1.5
Nipple discharge-dermatitis of the nipple	1	0.3
Infant’s ineffective attachment to the breast	17	4.3
Mother-related causes		
Choice	34	8.6
Medication intake	57	14.4
Smoking	11	2.8
Caffeine consumption	1	0.3
Return to work	12	3.0
Disease	6	1.5
Initiation of pregnancy	2	0.5
Difficulty in time management, Fatigue, Poor sense of self-efficacy	21	5.6
Bad breastfeeding experience	5	1.3
Infant-related causes		
Hospitalization	6	1.5
Poor weight gain	5	1.3
Gastroesophageal reflux	4	1.0
Jaundice	1	0.3
Infant’s preference to non-human milk	5	1.3
Possible allergy to dairy products	2	0.5
Total	397	100.0

**Table 4:** Breastfeeding indicators per time period (%). Comparison with National Study 2017 (ICH).

Infant’s Age	FBF (%)		BFWHMS (%)		ABF (%)	
	Τigka et al.	ΙCH	Τigka et al.	ΙCH	Τigka et al.	ΙCH
4 days ^a^/1 week ^b^	53.5	51.13	53.5	51.13	94.1	90.84
1 month	47.2	42.16	47.2	42.16	84.0	79.89
3 months	40.7	32.16	40.7	32.16	69.2	63.47
6 months	7.2	0.78	30.7	23.53	53.1	45.39

FBF: full breastfeeding; BFWHMS: breastfeeding without human-milk substitute; ABF: any breastfeeding; ICH: institute of child health; ^a^: concerns present study (Tigka et al.); ^b^: concerns National Study 2017 (ICH).

## Data Availability

Data sharing not applicable. No new data were created or analyzed in this study. Data sharing is not applicable to this article.

## Supplementary Materials

The following are available online at https://www.mdpi.com/article/10.3390/children9010043/s1, Table S1: Logistic regression results for FBF in the first month, Table S2: Logistic regression results for FBF in the third month, Table S3: Logistic regression results for FBF in the sixth month, Table S4: Logistic regression results for ABF in the sixth month, Table S5: Logistic regression results for BFWHMS in the sixth month, Table S6: Employment at six months in association with ABF, Table S7: Employment at six months in association with FBF, Table S8: Employment at six months in association with BFWHMS, Figure S1: Comparison between the present and the latest national study on breastfeeding indicators per time period.
