# Supplementary material for: Frequency and Determinants of Breastfeeding in Greece: A Prospective Cohort Study during the COVID-19 Pandemic
Source: Children (Basel). 2022 Jan 2;9(1):43. doi: 10.3390/children9010043 (PMC8774342; doi:10.3390/children9010043)
Supplement: Supplementary file 1 [file children-09-00043-s001.zip › children-1483963-supplementary.pdf]

Table S1: Logistic regression results for FBF in the first month. (Nagelkerke R^2=36.4)

Variables	O.R.	p-value	95% C.I. for O.R.	
			Lower	Upper
Employment at sixth month after delivery	5.922	<0.001	4.133	8.485
Type of hospital	0.686	0.028	0.490	0.960
Mode of delivery	0.561	0.001	0.395	0.796
BMI before pregnancy	0.940	<0.001	0.909	0.972
Junior High/High School	10.424	0.050	1.004	108.265
College	16.692	0.019	1.602	173.903
University	22.824	0.008	2.229	233.728
Postgraduate studies	17.590	0.017	1.681	184.041
Birth weight of the newborn <2500kg	0.257	0.005	0.100	0.662
Previous breastfeeding experience (days)	1.002	<0.001	1.002	1.003

Note: the reference level for education is none/elementary school.
FBF: full breastfeeding

Table S2: Logistic regression results for FBF in the third month. (Nagelkerke R^2=41.2)

Variables	O.R.	p-value	95% C.I. for O.R.	
			Lower	Upper
Previous breastfeeding experience (days)	1.002	<0.001	1.001	1.003
Employment at sixth month after delivery	12.864	<0.001	8.210	20.159
BMI before pregnancy	0.930	<0.001	0.897	0.963
Mode of delivery	0.585	0.004	0.407	0.840
Prematurity	0.481	0.069	0.219	1.059
Marital status	3.450	0.008	1.382	8.613
Birth weight of the newborn <2500kg	0.288	0.023	0.098	0.842

FBF: full breastfeeding

Table S3: Logistic regression results for FBF in the sixth month. (Nagelkerke R^2=23.4)

Variables	O.R.	p-value	95% C.I.for O.R.	
			Lower	Upper
Previous breastfeeding experience (days)	1.002	<0.001	1.002	1.003
Employment at sixth month after delivery	11.741	0.001	2.805	49.149

FBF: full breastfeeding

Table S4: Logistic regression results for ABF in the sixth month. (Nagelkerke R^2=65.7)

Variables	O.R.	p-value	95% C.I.for O.R.	
			Lower	Upper
Employment at sixth month after delivery	66.579	<0.001	38.189	116.073
BMI before pregnancy	0.948	0.012	0.909	0.988
Junior High/High School	45.636	0.004	3.370	617.953
College	54.590	0.003	4.008	743.598
University	109.635	<0.001	8.150	1474.902
Postgraduate studies	210.877	<0.001	14.874	2989.717

Previous breastfeeding experience (days)	1.004	<0.001	1.003	1.006

Note: the reference level for education is none/elementary school.

ABF: any breastfeeding

Table S5: Logistic regression results for BFWHMS in the sixth month. (Nagelkerke R^2=37.9)

Variables	O.R.	p-value	95% C.I.for O.R.	
			Lower	Upper
Employment at sixth month after delivery	31.595	<0.001	15.798	63.191
Type of hospital	0.542	0.001	0.383	0.767
Marital status	3.326	0.021	1.197	9.247
Mode of delivery	0.612	0.007	0.428	0.874
BMI before pregnancy	0.950	0.007	0.916	0.986
Birth weight of the newborn <2500kg	0.187	0.003	0.062	0.560

BFWHMS: breastfeeding without human-milk substitute

Table S6: Employment at six months in association with ABF.

		ABF		
		Women who followed ABF	Women who did not follow ABF	Total
Employment at	Yes	21	285	306
	No (Household/Unemployed)	99	72	171
	No (Maternity Leave)	127	25	152
	No (Work suspension due to COVID-19 pandemic)	83	2	85
	No (Teleworking due to COVID-19 pandemic)	124	8	132
Total		454	392	846

Chi-square test: p-value<0.001

ABF: any breastfeeding

Table S7: Employment at six months in association with FBF.

		FBF		
		Women who followed FBF	Women who did not follow FBF	Total
Employment at six months	Yes	2	304	306
	No (Household/Unemployed)	13	158	171
	No (Maternity Leave)	1	151	152
	No (Work suspension due to COVID-19 pandemic)	30	55	85
	No (Teleworking due to COVID-19 pandemic)	15	118	133
Total		61	786	847

Chi-square test: p-value<0.001

FBF: full breastfeeding

Table S8: Employment at six months in association with BFWHMS.

			BFWHMS		
			Women who followed BFWHMS	Women who did not follow BFWHMS	Total
Employment at six months	Yes	Count	9	297	306
	No (Household/Unemployed)	Count	62	109	171
	No (Maternity Leave)	Count	79	73	152
	No (Work suspension due to COVID-19 pandemic))	Count	35	50	85
	No (Teleworking due to COVID-19 pandemic)	Count	78	54	132
Total		Count	263	583	846
Chi-square test: p-value<0.001					

BFWHMS: breastfeeding without human-milk substitute

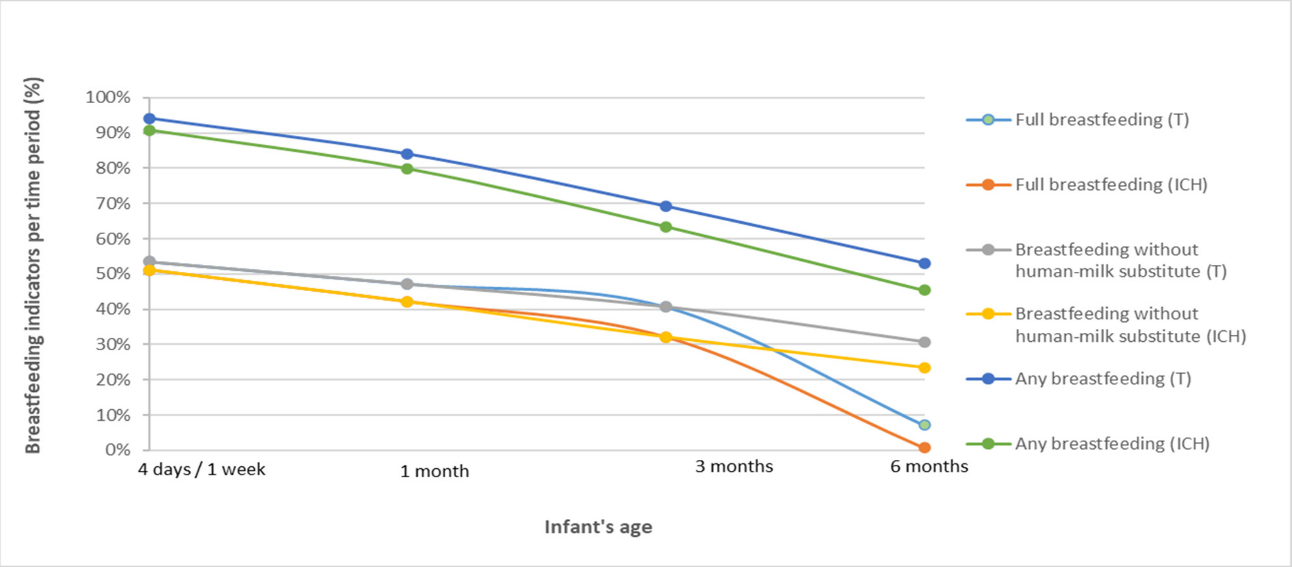

Figure S1. Comparison between the present and the latest national study on breastfeeding indicators per time period.

T: Tigka et al. (present study); ICH: Institute of Child Health (Latest National Study)
